# Modulation of Pilocarpine-Induced Seizures by Cannabinoid Receptor 1

**DOI:** 10.1371/journal.pone.0095922

**Published:** 2014-04-21

**Authors:** Rebecca L. Kow, Kelly Jiang, Alipi V. Naydenov, Joshua H. Le, Nephi Stella, Neil M. Nathanson

**Affiliations:** Department of Pharmacology, University of Washington, Seattle, Washington, United States of America; Dalhousie University, Canada

## Abstract

Administration of the muscarinic agonist pilocarpine is commonly used to induce seizures in rodents for the study of epilepsy. Activation of muscarinic receptors has been previously shown to increase the production of endocannabinoids in the brain. Endocannabinoids act at the cannabinoid CB_1_ receptors to reduce neurotransmitter release and the severity of seizures in several models of epilepsy. In this study, we determined the effect of CB_1_ receptor activity on the induction in mice of seizures by pilocarpine. We found that decreased activation of the CB_1_ receptor, either through genetic deletion of the receptor or treatment with a CB_1_ antagonist, increased pilocarpine seizure severity without modifying seizure-induced cell proliferation and cell death. These results indicate that endocannabinoids act at the CB_1_ receptor to modulate the severity of pilocarpine-induced seizures. Administration of a CB_1_ agonist produced characteristic CB_1_-dependent behavioral responses, but did not affect pilocarpine seizure severity. A possible explanation for the lack of effect of CB_1_ agonist administration on pilocarpine seizures, despite the effects of CB_1_ antagonist administration and CB_1_ gene deletion, is that muscarinic receptor-stimulated endocannabinoid production is acting maximally at CB_1_ receptors to modulate sensitivity to pilocarpine seizures.

## Introduction

Muscarinic acetylcholine receptors (mAChRs) mediate many of the actions of acetylcholine in the central nervous systems [1]. There are five mAChR subtypes, all of which are G protein-coupled receptors (GPCRs). The M_1_, M_3_, and M_5_ subtypes preferentially couple to members of the G_q/11_ family of G-proteins to activate phospholipase C-β (PLCβ), while the M_2_ and M_4_ subtypes preferentially couple to members of the G_i/o_ family to inhibit adenylyl cyclase [2]. In the brain, muscarinic receptors are involved in processes such as learning, memory, control of movement, nociception, temperature control, as well as in the modulation of signaling by other neurotransmitters [1], [3], [4]. The M_1_ subtype is the predominant mAChR in the forebrain with high expression in the hippocampus, cortex, and striatum [5], where it has been implicated in learning and memory [1], [5], [7], [8]. In addition, the M_1_ receptors mediates seizure induction due to administration of muscarinic agonists such as pilocarpine [6].

Pilocarpine is a muscarinic agonist commonly used to induce seizures in rodents because it produces a phenotype that resembles human temporal lobe epilepsy [9]. After recovery from the initial period of seizure activity, pilocarpine-treated animals develop spontaneous seizures a few weeks later. During this latent period prior to the development of spontaneous seizures, the brain, especially the hippocampus, undergoes many changes including increased cell proliferation, cell death and mossy fiber sprouting [10], [11]. Induction of pilocarpine seizures is blocked by pretreatment with muscarinic antagonists, but subsequent administration of muscarinic antagonists will not terminate seizure activity, indicating that muscarinic receptor activation is required for the induction of seizures but is not required for their maintenance [12], [13].

Endogenous cannabinoids (endocannabinoids, eCB) and CB_1_ receptors agonists have anticonvulsant activity in the electroshock seizure, the spontaneous seizure, and the kainic acid seizure models of epilepsy, while CB_1_ antagonists have proconvulsive activity in these models [14], [15], [16], [17]. CB_1_ receptors couple to G_i/o_ proteins and are predominantly located on presynaptic nerve terminals [18]. Activation of CB_1_ receptors serves as a feedback mechanism to modulate neurotransmitter signaling. The activity-dependent production and release of eCBs from postsynaptic cells leads to the activation of presynaptic CB_1_ receptors that inhibit neurotransmitter release [19]. eCB production is increased by either electrical activity or activation of GPCRs such as the M_1_ and M_3_ receptors that couple to PLCβ, the enzyme involved in the production of 2-arachidonoylglycerol, the most abundant eCB [20], [21], [22].

Previous studies on the role of CB_1_ receptors in the pilocarpine model of epilepsy have focused on the role of this receptor after the induction of seizures [15], [23], [24], [25]. These studies examined the role of CB_1_ receptors during the latent phase following the initial seizures or the chronic phase of spontaneous seizures, which is well past the time when muscarinic receptor activity is necessary for seizures. Therefore, in order to examine the effect of CB_1_ receptor activity on the induction of seizures by activation of muscarinic receptors, we determined the effects of administration of CB_1_ receptor agonists and antagonists and of the deletion of the CB_1_ receptor gene on the induction of pilocarpine-induced seizures.

## Materials and Methods

### Animals

All procedures involving animals were approved by the University of Washington Institutional Animal Care and Use Committee under protocols #2239-01 and #3233-05. CB_1_ knockout (KO) mice were obtained from Dr. Giovanni Marsicano [26] and were bred at the University of Washington. C57Bl/6 male mice were obtained from Charles River and used at 13 weeks of age.

### Drugs

Pilocarpine hydrochloride was purchased from Sigma Aldrich and dissolved in 0.9% saline. Diazepam (Hospira) and phenobarbitol (West-Ward) were purchased as stock solutions dissolved in 0.9% saline from the University of Washington Medical Center Pharmacy. SR141716 was obtained from the NIDA Drug Supply Program and was prepared in pharmasolve/cremophor RH40 (pharmasolve: cremophor RH40: drug, 1∶9:40). CP55940 was obtained from the NIDA Drug Supply Program and was prepared in a vehicle solution consisting of cremophor RH40: ethanol: saline (1∶1:18).

### Behavioral Responses to CP55940

Adult male WT or CB_1_ KO mice on a C57Bl/6 background were injected i.p. with 0.3 mg/kg CP55940, and tetrad behaviors were measured as described by Martin et al. [27]. Core temperature was measured using an anal probe. Locomotion was videotaped over 10 minutes in an Open Field chamber (45 cm×25 cm×25 cm), with Noldus Ethovision (Wageningen, the Netherlands). Catalepsy was scored by measuring latency to remove forepaws from a bar placed 3 cm above the bench surface, with a maximum score of 30s. For each animal, three trials were performed, and the trial with the longest latency was recorded. Analgesia was measured by tail flick; tails were immersed up to one cm in 52°C–56°C water bath, and latency to withdraw the tail was measured, up to a maximum time of 15 seconds. Tails were always inspected for tissue damage immediately after the experiment, and monitored the next day.

### Seizures

To compare seizure severity after pilocarpine treatment, mice were observed for 1 hour and scored as previously described [6] by an observer blind to drug treatment or genotype. In this scale, 1: tremor; 2: single myoclonic jerks; 3: clonus; 4: one tonic-clonic seizure; 5∶2 seizures; 6∶3 or more seizures, death, or *status epilepticus* (SE).

To examine cell proliferation and cell death after pilocarpine-induced SE, mice were injected with 225–250 mg/kg pilocarpine. After 2 hours of SE or 2.5 hours after pilocarpine administration if no SE occurred, mice were injected with 4 mg/kg diazepam to reduce excitability. Forty five minutes later, 30 mg/kg phenobarbitol was given to maintain the blockade of residual seizure activity.

### Tissue Processing

Four days after pilocarpine treatment, mice were treated with 140 mg/kg ketamine/10 mg/kg xylazine, perfused, and fixed with 4% paraformaldehyde in 0.1 M phosphate buffer, pH 7.4, overnight. After brains were soaked in 30% sucrose in PBS, they were frozen on dry ice. Frozen brains were sectioned at 30 µm with a cryostat and sections were stored at −20°C in a cryoprotectant solution (30% ethylene glycol, 30% glycerol, 0.1 M phosphate buffer, pH 7.4).

### Proliferating Cell Nuclear Antigen (PCNA) Immunofluorescence

Thirty µm free-floating sections were washed in PBS and PBST (PBS containing 2% Triton X-100) before antigen retrieval (10 minutes of boiling in 10 mM sodium citrate, pH 6). Sections were blocked in blocking buffer (2% bovine serum albumin, 0.1 M glycine, 0.05% sodium azide in PBST) plus 10% donkey serum for 1 hour at room temperature before overnight incubation at 4°C in 1∶1000 mouse anti-PCNA (Santa Cruz) in blocking solution. After multiple PBST washes, sections were incubated in 1∶500 donkey anti-mouse IgG Alexa Fluor 488 (Invitrogen) for 3 hours at room temperature. Sections were counterstained with 10 µM Hoechst 33342 in PBS for 10 minutes and mounted with Vectashield (Vector Laboratories).

### Fluoro-jade B (FJ) Staining

FJ staining was performed as described by the manufacturer (Millipore). Thirty µm sections were mounted on gelatin-subbed slides and allowed to dry at room temperature overnight. The next day, slides were incubated for 5 minutes in 1% sodium hydroxide in 80% ethanol, 2 minutes in 70% ethanol, and 2 minutes in water. Slides were then incubated for 10 minutes in 0.06% potassium permanganate and 2 minutes in water prior to FJ staining (0.0004% in 0.1% acetic acid for 20 minutes). After 3 washes with water, the slides were allowed to dry at room temperature overnight before coverslipped with DPX mountant for histology (Sigma Aldrich).

### Quantification

Images of hippocampal tissue were taken with a 10× objective on a Nikon Eclispe S600 equipped with a QImagine QIClick camera. Positively-stained cells were counted in the hilus from at least 8 sections from each animal. Counting was done blind to treatment using ImageJ (NIH). The total number of positive cells per animal was normalized to the total hilus area measured per animal.

### Data Analysis

Seizure severity scores and imaging data are presented as medians ± upper and lower quartiles. The Mann-Whitney U test was used to test the significance of seizure severity scores. The Fisher’s exact test was used for fractions of mice experiencing seizures. The Kruskal Wallis test was used for the quantifications of PCNA- and Fluoro-jade B-positive cells, with post-hoc Bonferroni-corrected Mann-Whitney U tests performed between groups. Cannabinoid tetrad scores are presented as the means ± SEM. Fisher’s T-test was used for analysis of cannabinoid tetrad behaviors. P values of less than 0.05 were considered statistically significant.

## Results

### Loss of CB_1_ Receptor Activity Increases Pilocarpine Seizure Severity

**Figure 1:**
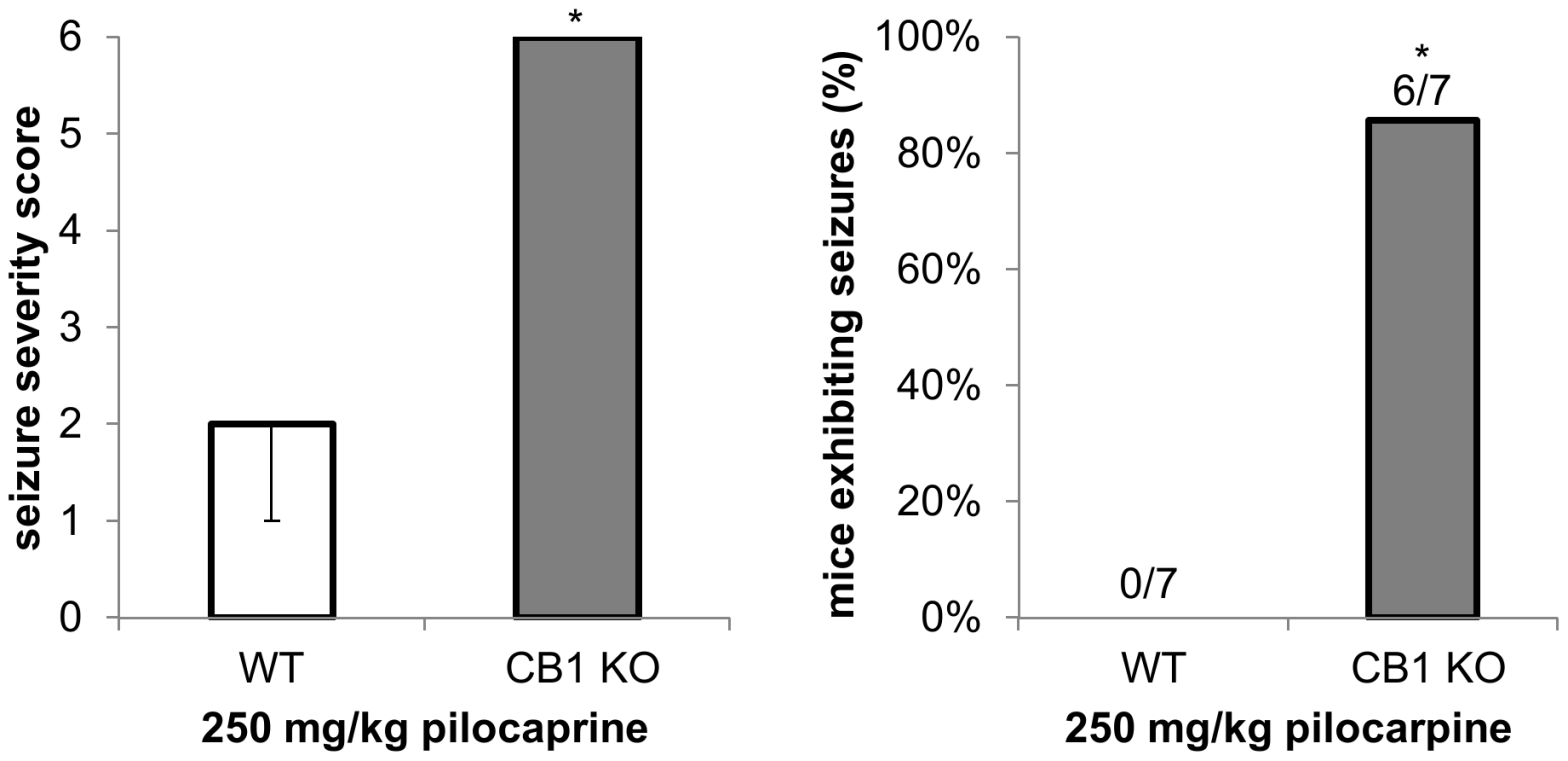
CB_1_ KO mice are more sensitive to pilocarpine. Seizure severity scores and the proportion of mice having at least one clonic-tonic seizure after injection with 250 mg/kg pilocarpine were compared in male CB_1_ KO (n = 7) and WT (n = 7) littermates. * p<0.05; ** p<0.005. Data are presented as medians ± upper and lower quartiles.

To investigate the role of the CB_1_ receptor in the induction of pilocarpine seizures, we first compared seizure severity after pilocarpine treatment in WT and CB_1_ KO mice. Using a submaximal dose of pilocarpine (250 mg/kg), we observed more severe seizure behaviors in CB_1_ KO mice than WT mice (Fig. 1). Both the average seizure severity score and the fraction of mice having full seizures (at least one tonic-clonic seizure) were significantly higher in CB_1_ KO mice compared to WT mice.

**Figure 2:**
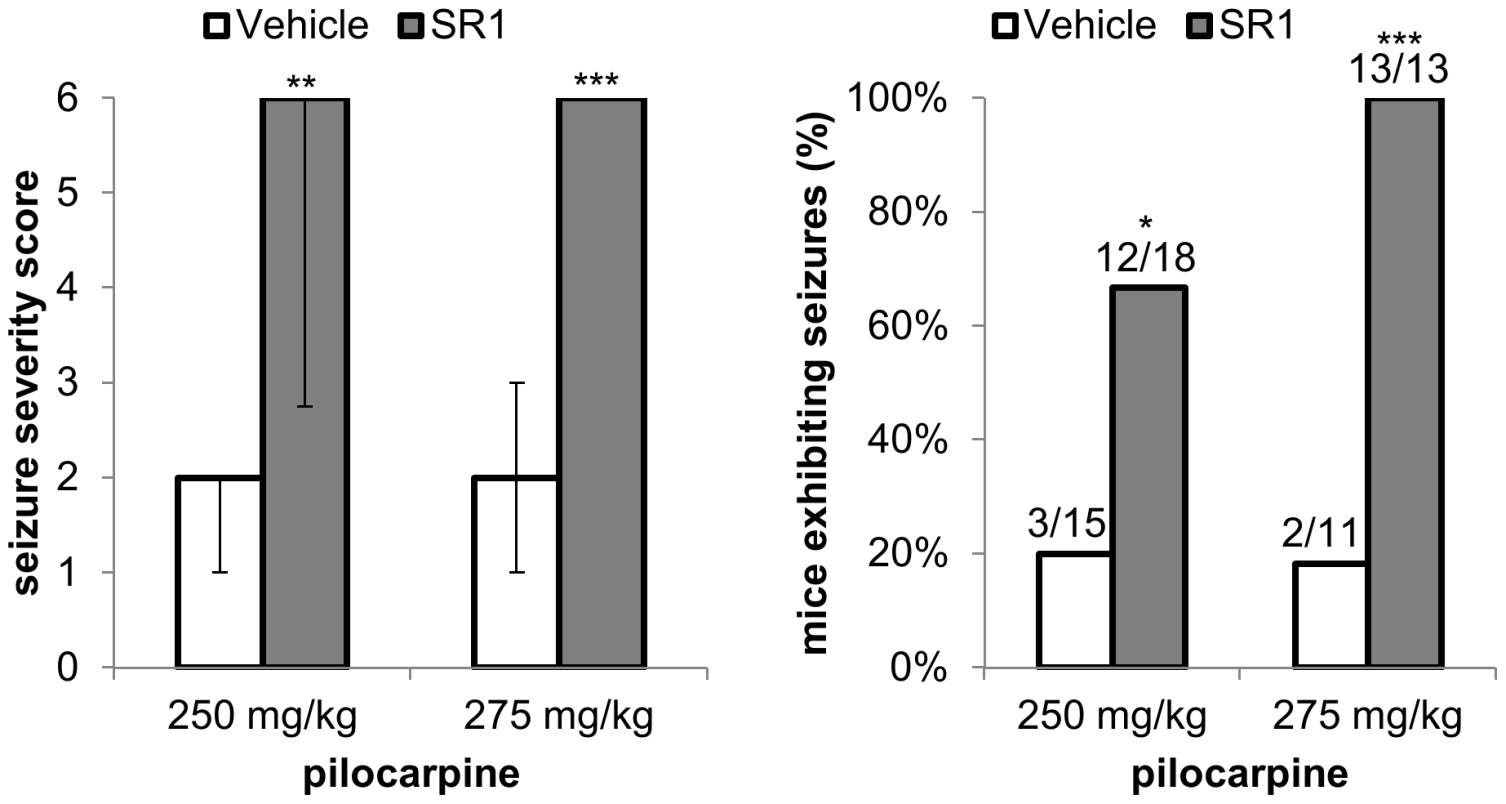
CB_1_ receptor antagonist pretreatment increases pilocarpine seizure sensitivity. SR141716 (SR1, 10 mg/kg) or the corresponding vehicle were given 2 hours prior to injection of 250 or 275 mg/kg pilocarpine. Seizure severity scores and the proportion of mice exhibiting at least one tonic-clonic seizure were compared between SR1 (250 mg/kg, n = 18; 275 mg/kg, n = 13) and vehicle-treated mice (250 mg/kg, n = 15; 275 mg/kg, n = 11). * p<0.05; ** p<0.005; *** p<0.0001. Data are presented as medians ± upper and lower quartiles.

To ensure that the increased sensitivity to pilocarpine-induced seizures in CB_1_ KO mice was due to loss of CB_1_ receptor activity and not due to a developmental or compensatory difference caused by a lack of CB_1_ receptors from birth, we compared pilocarpine-induced seizures in mice pretreated with the CB_1_ receptor antagonist SR141716 (SR1) or vehicle. At submaximal doses of pilocarpine (250 and 275 mg/kg), we saw an increase in the severity of seizure behaviors and in the proportion of mice experiencing full seizures with SR1 pretreatment (Fig. 2). This indicates that activity of the CB_1_ receptors, presumably due to the actions of endogenously released eCBs, modulates the sensitivity to seizure induction by pilocarpine.

### Pilocarpine Seizure-induced Increases in Cell Proliferation and Cell Death are Unchanged by CB_1_ Antagonist

Pilocarpine-induced seizures, specifically pilocarpine-induced *status epilepticus* (SE), cause significant increases in both cell proliferation and cell death in the brain days following the initial seizures [10], [11]. To determine whether blockade of CB_1_ receptor activity led to any changes in seizure-induced cell proliferation and cell death, we performed immunohistochemistry focusing on the hilus 4 days after pilocarpine treatment.

**Figure 3:**
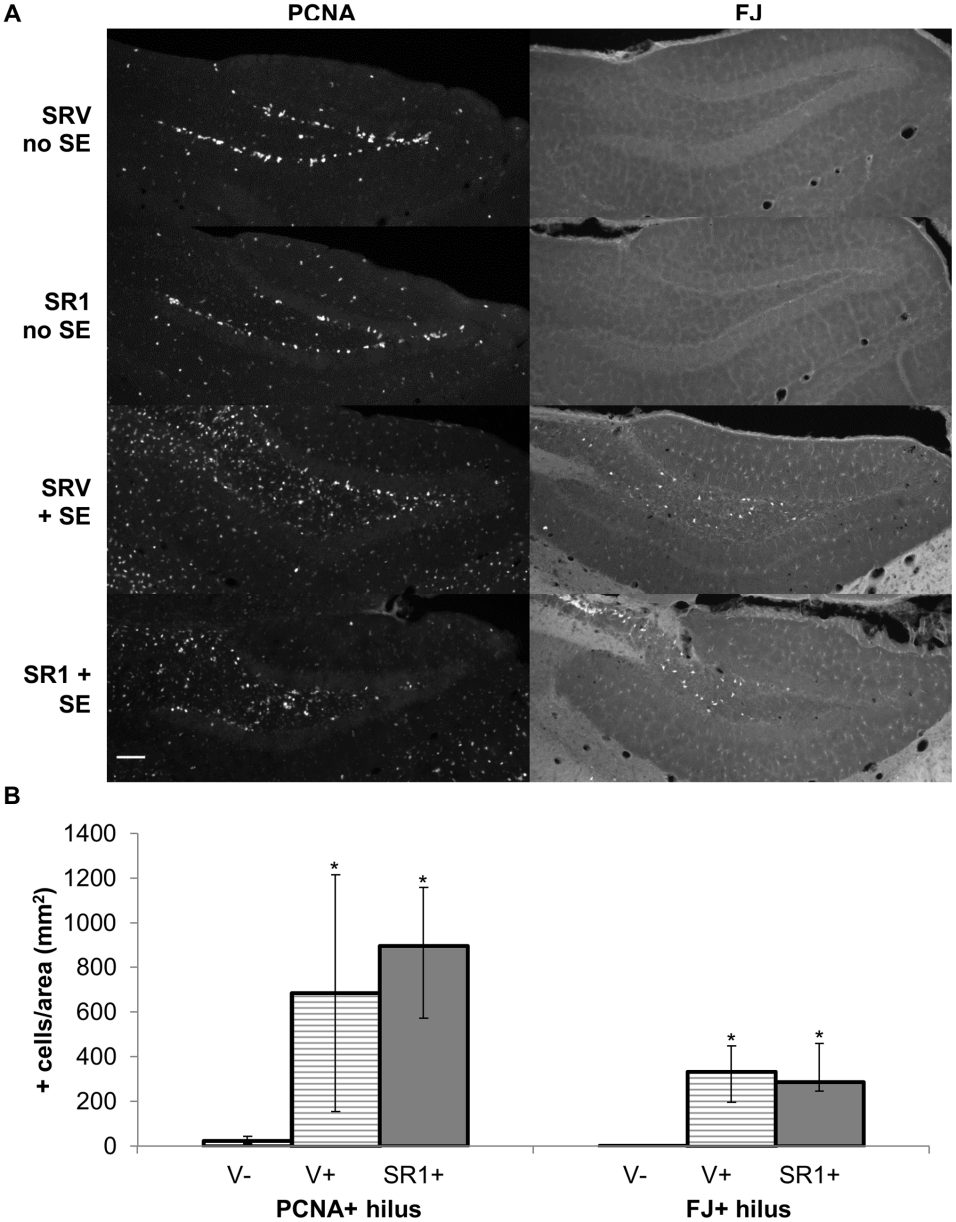
Cell death and cell proliferation after pilocarpine-induced SE are unchanged by SR1 pretreatment. Mice were pretreated for 2 hours with SR141716 (10 mg/kg) or vehicle before injection with 225–250 mg/kg pilocarpine. Brains were harvested for immunohistochemistry analysis 4 days after pilocarpine treatment. **A.** Representative images of proliferating cell nuclear antigen (PCNA) immunofluorescence and Fluoro-jade B (FJ) staining from (top to bottom) vehicle-treated mice without SE (V−), SR1-treated mice without SE (SR1−), vehicle-treated mice with SE (V+), and SR1-treated mice with SE (SR1+). Scale bar = 100 µm. **B.** PCNA and FJ quantifications in the hilus of vehicle-treated mice without SE (V−, n = 6), vehicle-treated mice with SE (V+, n = 3), and SR1-treated mice with SE (SR1+, n = 5). The number of positive cells was normalized to the area of hilus measured *per* animal. * p<0.05 when compared to vehicle-treated mice without SE (V−). Data are presented as medians ± upper and lower quartiles.

We used proliferating cell nuclear antigen (PCNA), a protein commonly used as an endogenous marker of proliferation [28], to measure seizure- and/or drug-induced cell proliferation. PCNA immunoreactivity was greatly increased throughout the hippocampus of mice that experienced SE, as previously reported [11], [29], while it remained mostly limited to the subgranular zone of the dentate gyrus of mice that did not experience SE (Fig. 3A). Since variations in PCNA immunoreactivity in the subgranular zone could also be due to differences in basal neurogenesis, we quantified PCNA immunoreactivity in the hilus, a region that normally does not have significant amounts of cell proliferation in adult mice. Quantification of PCNA-immunopositive cells in the hilus showed a significant increase in cell proliferation in mice that experienced SE compared to those that did not (Fig. 3B). SR1 pretreatment did not modify the amount of cell proliferation induced by SE, indicating that seizure activity itself caused the increases in cell proliferation.

To measure changes in cell death, we stained sections with Fluoro-jade B, which labels degenerating cells [30]. Fluoro-jade B staining showed a significant increase in degenerating cells in the hilus of mice that experienced SE and this response was not altered by SR1-pretreatment (Fig. 3A and 3B).

In line with our result showing that SR1 pretreatment increased the probability and the severity of pilocarpine seizures, only 1 out of the 22 SR1-pretreated mice did not experience SE or death. The amount of cell proliferation and cell death in the hilus of this mouse was similar to vehicle-pretreated mice that did not experience SE (PCNA-positive: 36 cells/mm^2^ versus 27±8 cells/mm^2^; FJ-positive: 1.6 cells/mm^2^ versus 0.8±0.3 cells/mm^2^). Together the results indicate that CB_1_ receptor antagonism itself does not increase cell proliferation or cell death but rather that the increases in cell proliferation and cell death are due to the increases in pilocarpine seizure severity measured in mice pretreated with the CB_1_ receptor antagonist.

### Treatment with CB1 agonist does not Alter Pilocarpine Seizure Severity

**Figure 4:**
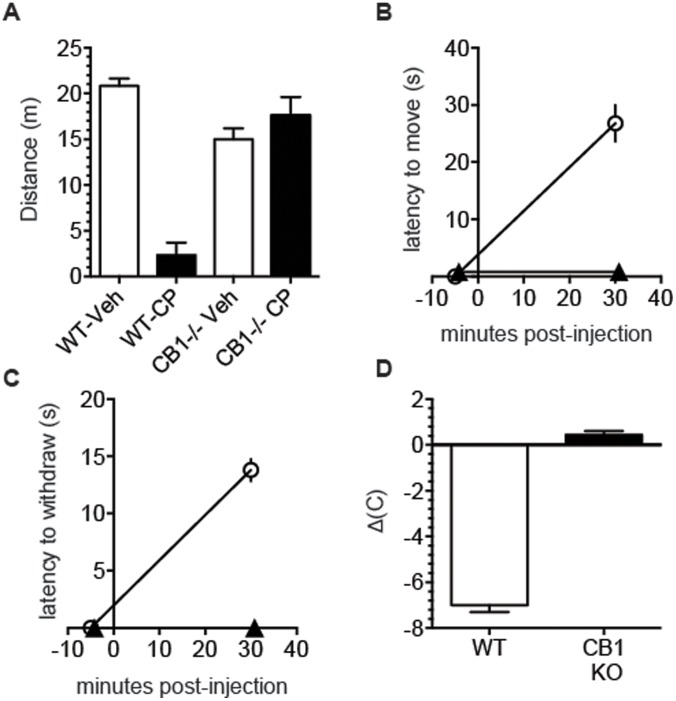
Administration of CP55940 results in CB1-dependent cannabinoid response. Cannabinoid tetrad behaviors (hypolocomotion, catalepsy, analgesia, and hypothermia) were measured after i.p. administration with 0.3 mg/kg CP in WT (n = 5) and CB_1_-KO mice (n = 5). **A.** Mice were treated with either vehicle or CP, and placed in an open-field chamber 30 minutes after treatment. **B.** Catalepsy was measured by the bar test either 5 minutes before, or 30 minutes after CP treatment in WT and CB_1_ KO mice. **C.** Analgesia was measured by tail flick both before and after CP treatment. **D.** Hypothermia was measured by comparing core body temperature before and 30 minutes after treatment with CP. Data are presented as means ± SEM. ***p<0.001.

The results above indicate that blockade or genetic deletion of CB_1_ receptors increases the severity of pilocarpine-induced seizures, most likely due to elimination of the action of eCB at the CB_1_ receptors. In order to test if pretreatment with a CB_1_ receptor agonist affected pilocarpine –induced seizures, we first ensured that pretreatment with the CB_1_ receptor agonist CP55940 (CP) would elicit CB_1_- mediated responses in mice. Administration of 0.3 mg/kg CP (i.p.), resulted in characteristic behavioral effects in WT, but not CB_1_ KO mice. Locomotor activity in an open field chamber (Fig. 4A) was reduced in WT but not CB_1_ KO mice. Similarly, although no mice demonstrated any measureable catalepsy prior to treatment (Fig. 4B), 30 minutes after administration of CP, WT mice developed catalepsy whereas CB_1_ KO mice showed no change. CP-induced hypothermia was measured by comparing the core body temperature 5 minutes before, and 30 minutes after drug administration (Fig. 4D). WT mice developed marked hypothermia, whereas CB_1_ KO mice were unaffected (WT: ΔT = −7.0±0.3°C; CB_1_ KO: ΔT = 0.4±0.9°C). Finally, analgesia was measured by the tail flick method (Fig. 4C). Prior to CP administration, all animals removed their tails immediately upon immersion, but 30 minutes after drug administration, WT mice took 13.8±1.0 s to remove their tails, whereas CB_1_ KO mice continued to remove their tails immediately upon immersion. Based on these results, we confirm previous studies [31], [32] showing that 0.3 mg/kg CP administered i.p. results in hypolocomotion, catalepsy, hypothermia, and analgesia in WT but not CB_1_ KO mice.

**Figure 5:**
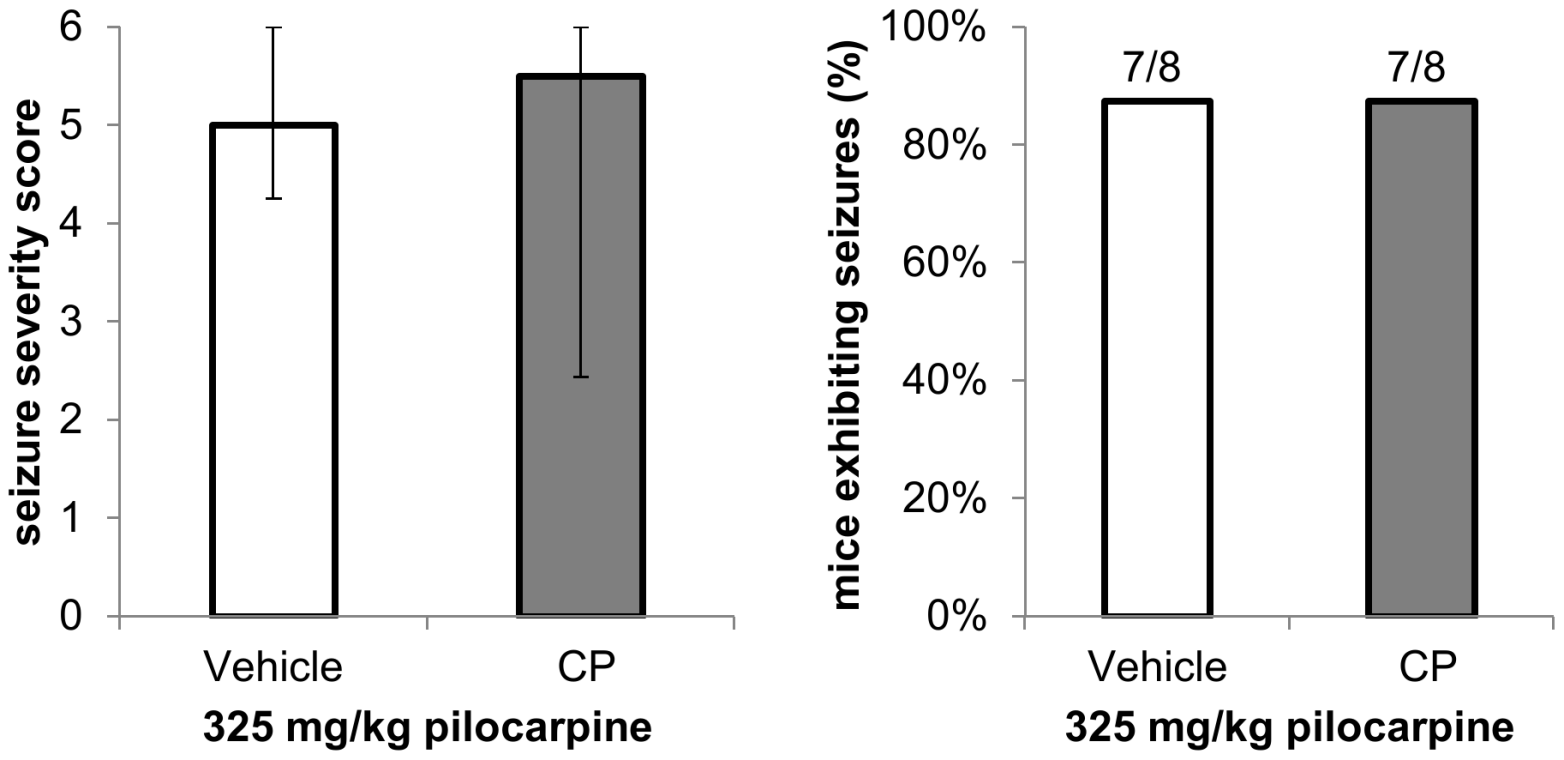
CB_1_ receptor agonist pretreatment does not reduce pilocarpine seizure severity. Mice were pretreated with CP 55940 (CP, 0.3 mg/kg) or vehicle 30 minutes prior to pilocarpine (325 mg/kg). Seizure severity scores and the proportion of mice having at least one clonic-tonic seizure were compared between CP-treated (n = 8) and vehicle-treated mice (n = 8). Data are presented as medians ± upper and lower quartiles.

We then tested whether pretreatment with CP reduces pilocarpine-induced seizure severity. We found no difference in seizure severity scores or in the proportion of mice that exhibited clonic-tonic seizures between CP- and vehicle-pretreated mice in response to administration of 325 mg/kg pilocarpine (Fig. 5). Previous studies have shown that stimulation of M_1_ and M_3_ muscarinic receptors in the hippocampus stimulates eCB production *via* the activation of PLCβ [20], [21], [22]. The lack of effect of CP administration on pilocarpine-induced seizures suggests that eCB activity at the CB_1_ receptors is already at maximal levels following high dose pilocarpine administration, so administration of exogenous CB_1_ receptor agonist cannot further modulate the sensitivity to pilocarpine seizures.

## Discussion

Activation of CB_1_ receptors has been shown to reduce the severity of seizures in a variety of models of epilepsy, and decreased activation of CB_1_ receptors increases the severity of spontaneous, kainic acid-induced, and electroshock-induced seizures [14], [15], [16]. We show here that the loss of CB_1_ receptor activity, due to genetic deletion in knockout animals or by administration of CB_1_ receptor antagonists, causes an increased sensitivity to pilocarpine-induced seizures. Previous studies have shown that activation of M_1_ and M_3_ receptors stimulate the production of endocannabinoids [20], [21]. The increased sensitivity to pilocarpine-induced seizures following administration of a CB_1_ antagonist and when the CB_1_ gene is deleted indicates that eCBs acting at the CB_1_ receptor modulate the severity of pilocarpine-induced seizures.

Induction of SE causes many changes in the brain, including increased neurogenesis, gliosis, mossy fiber sprouting, and cell death [10], [11]. We confirmed that pilocarpine-induced SE caused the expected increases in cell proliferation and cell death in the hilus in mice that had experienced SE. SE-induced damage was not modified by the loss of CB_1_ receptor activity during the seizure period, suggesting that changes in cell proliferation and cell death are not due directly to CB_1_ receptor activity but rather reflect the effect of CB_1_ receptor activity on seizure severity and ensuing cell proliferation and cell death. Previous studies using kainic acid reported that loss of CB_1_ receptor activity caused a decrease in neurogenesis and an increase in cell death and gliosis [16], [33]. In the study by Aguado et al. [33], 10 µM kainic acid was sufficient to significantly increase neurogenesis in a CB_1_-dependent manner *in vitro*. However, when tested *in vivo*, Aguado et al. did not indicate whether the dose of kainic acid used (15 mg/kg) in WT and CB1 KO mice evoked seizures or the severity of any seizures evoked. Therefore the decrease in neurogenesis due to loss of CB_1_ receptor activity in their study may be mostly or entirely seizure-independent.

Marsicano et al. [16] also did not indicate whether the quantification of cell death in their study was performed on mice that had experienced SE or not. Because the dose of kainic acid that they used (20 mg/kg) typically caused more severe seizures in CB_1_ KO mice compared to WT mice, they may have compared cell death between WT mice that did not have SE to CB_1_ KO mice that did have SE. If this was the case, their results are consistent with the fact that CB_1_ KO mice are more sensitive to kainic acid-induced seizures but do not indicate whether or not CB_1_ activity modified kainic acid-induced seizure damage.

While we and others found that loss of CB_1_ receptor activity increased seizure incidence and severity, we saw no effect of CB_1_ receptor agonist pretreatment on the sensitivity or severity of pilocarpine-induced seizures. This resistance to exogenous CB_1_ agonist administration suggests that CB_1_ receptor activity was already maximally modulating pilocarpine seizures *via* the actions of eCBs. In contrast, increasing CB_1_ receptor activity reduced the severity of electroshock, spontaneous, and kainic acid-induced seizures [14], [15], [16]. CB_1_ receptor agonists reduced or abolished seizures in the electroshock and spontaneous seizure model [14], [15], while the eCB reuptake inhibitor UCM707 reduced the severity of kainic acid seizures [16]. This suggests that eCB production may differ between models of seizure induction.

Kainic acid, pilocarpine, and seizure activity itself have been shown to increase eCB levels, but the identity and the quantity of the eCB increased have yet to be fully determined. While anandamide but not 2-AG was increased after kainic acid seizures [16], only changes in 2-AG were measured after pilocarpine seizures [15]. Future comparisons of changes in eCB levels in response to kainic acid or pilocarpine administration may allow determination of whether differences in drug- or seizure-induced eCB levels account for differences in the anticonvulsive activity of exogenously applied CB_1_ receptor agonists.

The extent of change in the expression level of CB_1_ receptors over the course of epilepsy development is remarkably different depending on the models that are studied. While there are some discrepancies between studies looking at CB_1_ receptor expression following pilocarpine seizures, CB_1_ receptor expression decreases shortly after seizures and increases in epileptic mice [23], [24], [25], [34]. Specifically, CB_1_ receptor expression on presynaptic GABAergic terminals increased [34]. A short-term decrease in CB_1_ receptor expression could be due to receptor internalization, while long-term changes could be due to a compensatory mechanism [25]. Interestingly, a decrease in CB_1_ receptor expression is seen in human temporal lobe epilepsy patients [35]. A comparable analysis of CB_1_ receptor expression levels following kainic acid seizures could indicate whether differences in CB_1_ receptor expression levels correlates with differences in the anticonvulsive activity of CB_1_ activators in different epilepsy models.

In conclusion, we have demonstrated a role for CB_1_ receptors in the induction phase of pilocarpine-induced seizures. While loss of CB_1_ receptor activity changed the outcome of subthreshold pilocarpine-induced seizures, administration of a CB_1_ receptor agonist did not alter the severity of pilocarpine-induced seizures, suggesting that the ability of CB_1_ receptor to reduce pilocarpine-induced seizure severity is already maximized by muscarinic receptor-induced eCB. Further investigation of the effects of the CB_1_ receptor in this and other seizure models will expand our understanding on the actions and limitations of CB_1_ receptors in the regulation of seizures.
